# Effectiveness of an urban forest healing program for improving sleep in cancer survivors

**DOI:** 10.1016/j.apjon.2025.100795

**Published:** 2025-09-29

**Authors:** Sang Yi Baek, Kwang-Hi Park, Haneul Lee, Ji Hyun Sung, Min Kyung Song, Eun Young Park

**Affiliations:** a College of Nursing, Gachon University, Incheon, Republic of Korea; b Department of Physical Therapy, College of Medical Science, Gachon University, Incheon, Republic of Korea; c College of Nursing, Keimyung University, Daegu, Republic of Korea; d Department of Nursing, University of Ulsan, Ulsan, Republic of Korea

**Keywords:** Cancer survivor, Quality of life, Sleep, Forest therapy, Nature-based intervention

## Abstract

**Objective:**

Sleep disturbance is common among cancer survivors, impairing their psychological and physical well-being. While the effectiveness of cognitive behavioral therapy and exercise has been established, structured nature-based interventions remain underexplored. Forest therapy may improve sleep and health; however, data on its benefits in cancer survivors who experience sleep disturbances remain limited. This study aimed to evaluate the effectiveness of an eight-session structured forest therapy program in improving sleep among cancer survivors.

**Methods:**

A mixed-methods design was employed to integrate both quantitative and qualitative analyses. The study used a one-group pre–post-test design involving 29 cancer survivors who had completed curative treatments. The participants attended 8 structured 2-h sessions, held weekly in 2 urban forests. Sleep quality and experience, insomnia severity, sleep hygiene, psychological distress, fatigue, quality of life, muscle strength, and balance before and after the intervention were assessed. Qualitative data were collected through in-depth interviews and analyzed using qualitative content analysis.

**Results:**

All 29 participants reported significant improvements in sleep onset time, sleep quality, post-wake behavior, sleep hygiene, and insomnia severity (*P* ​< ​0.001). Psychological distress, cancer-related fatigue, and quality of life were also improved (*P* ​< ​0.001). Physical function indicators, including grip strength, balance, and cardiorespiratory endurance, were significantly improved as well (*P* ​< ​0.05). The qualitative findings revealed four themes: perceived forest benefits, empathy through shared experiences, active health self-management, and expanded self-awareness.

**Conclusions:**

Our structured forest therapy program is associated with improved sleep and well-being among cancer survivors, offering a promising non-pharmacological approach to survivorship care.

## Introduction

Cancer survival rates have significantly increased owing to advances in both early detection and treatment,^1^ with the global cancer incidence projected to reach 28.4 million by 2040.^2^ However, although extended survival is a promising outcome, many survivors experience lingering symptoms such as pain, fatigue, and, notably, sleep disturbances. A recent systematic review and meta-analysis reported that sleep disturbances were prevalent in 13.3–93.1% of cancer survivors, with a pooled prevalence of approximately 60.7%.^3^ Sleep disturbances impair physical recovery and negatively affect emotional well-being and quality of life, highlighting the importance of sleep as a crucial yet often overlooked aspect of care among cancer survivors.^3^^,^^4^

Sleep disturbances in cancer survivors are influenced by a combination of the physical side effects of treatment, persistent psychological distress, and maladaptive sleep patterns that develop during illness.^5^ Studies have shown that disruptions in circadian rhythm, heightened arousal, and changes in cortisol and melatonin levels can contribute to chronic sleep problems in this population.^6^ Sleep deprivation, in turn, exacerbates fatigue, depression, and immune dysfunction, further complicating posttreatment recovery.^7^^,^^8^

Conventional approaches for improving sleep in cancer survivors include cognitive behavioral therapy (CBT), exercise interventions, and mindfulness-based stress reduction, each of which has shown efficacy**.**^9^, ^10^, ^11^, ^12^, ^13^, ^14^ However, barriers such as access, cost, and time commitment often limit participation, particularly in outpatient settings.^15^ Recent reviews have highlighted an increasing preference of cancer survivors for sustainable, non-pharmacological interventions that are cost-effective, accessible, and compatible with their physical and emotional needs.^15^^,^^16^

In line with these preferences, nature-based interventions, such as forest therapy, have emerged as promising holistic approaches that integrate the physical, psychological, and environmental dimensions of health.^17^^,^^18^ Forest therapy typically includes guided walks, breathing exercises, and sensory engagement in natural environments, all of which are associated with reductions in stress, anxiety, and depressive symptoms. They also improve mood, sleep quality, and immune marker (e.g., cortisol) levels.^19^, ^20^, ^21^, ^22^, ^23^ These interventions also align well with cancer survivors’ preferences for low-burden, self-paced, and community-based programs.^24^

However, despite these promising outcomes, current evidence has several limitations. Most forest-based studies in cancer populations have broadly focused on emotional well-being or fatigue, with relatively few studies targeting sleep as a primary outcome.^19^, ^20^, ^21^^,^^25^ Additionally, many interventions were conducted in remote natural forests, posing accessibility challenges for survivors residing in urban areas.^24^ Furthermore, methodological limitations—such as small sample sizes, lack of control groups, and short follow-up durations—have constrained the generalizability of previous findings.^16^ These limitations are also present in the current study. However, unlike earlier research, our study emphasizes the ongoing need for further investigation by specifically addressing sleep as a primary outcome and evaluating a structured, accessible intervention for cancer survivors in urban environments. In this context, sleep was conceptualized from a multidimensional perspective, encompassing subjective sleep quality and experience, insomnia severity, and sleep hygiene.

We developed and evaluated a structured forest therapy program tailored to improve sleep among cancer survivors. The program was implemented in accessible urban forests and incorporated evidence-based components known to affect sleep-related behaviors and physiological responses; these included physical activity, mindfulness, and cognitive strategies.^26^^,^^27^ The structure and objectives of the program were informed by core principles of CBT for insomnia (CBT-I) and environmental restoration theory (ERT), both of which emphasize behavioral regulation and the role of restorative natural environments in promoting sleep recovery.^28^^,^^29^

Building on this conceptual framework, the present study employed a mixed-methods design to assess quantitative changes related to the multidimensional nature of sleep, psychological distress, and physical functioning while also collecting qualitative insights into the participants’ subjective experiences with the intervention. This comprehensive design enabled a more nuanced understanding of the potential benefits of structured forest therapy in cancer survivors with sleep disturbances. As a preliminary investigation, this study examined the feasibility and perceived value of implementing this intervention in real-world urban settings. The findings provide empirical support for integrating nature-based, non-pharmacological approaches into survivorship care and serve as a foundational reference for the future development of accessible and sustainable programs.^30^^,^^31^

## Methods

### Study design

This exploratory study aimed to evaluate the feasibility and preliminary effects of a forest therapy program in real-world clinical settings. An embedded mixed-methods^32^ design with a one-group pre-test–post-test structure was employed. A single-group design was selected because of the ethical and practical constraints in recruiting a comparison group that would be denied access to a potentially beneficial intervention.

### Participants and data collection

Cancer survivors who had completed curative treatments (surgery, chemotherapy, or radiotherapy) 3 months to 5 years prior to recruitment were eligible. The inclusion criteria were as follows: (1) age ≥ 20 years, (2) an Eastern Cooperative Oncology Group (ECOG)^33^ Performance Status score of 0 or 1, (3) sleep satisfaction score of ≤ 5 on a 10-point scale, and (4) not using sleep medications. The participants were recruited from the Cancer Survivorship Support Center and affiliated medical institutions between July 1 and August 20, 2022 using both online and offline materials. Of the 92 individuals screened, 31 individuals were eligible. After 2 participants withdrew due to surgery (*n* ​= ​1) and personal reasons (*n* ​= ​1), 29 participants were finally included.

### Procedure

Following IRB approval, recruitment requests were sent to the Cancer Survivorship Support Center and affiliated medical institutions, and posts were shared in online communities for cancer survivors. After obtaining written informed consent, the participants underwent physiological, physical, and psychological assessments using research-approved instruments approximately 2–3 days before the start of the forest therapy program.

The forest therapy program was conducted weekly for 8 weeks from September to November 2022 in 2 urban forests (Ansan and Incheon Grand Park). Post-intervention assessments were conducted twice via an online self-reported survey: within 3 days of program completion (including the same physiological, physical, and psychological measures and qualitative interviews) and 4 weeks thereafter. To ensure the authenticity of the follow-up survey responses, individualized survey links were sent via private mobile messages, and the participants were asked to provide identifying information during the online survey. This information was used solely for identity verification and was anonymized prior to analysis.

Three trained researchers conducted all assessments, while a certified forest therapy instructor and a trained facilitator led all sessions to ensure consistency and quality.

### Qualitative interviews

As part of the post-intervention assessments, semi-structured interviews were conducted to explore the participants' experiences with the forest therapy program. The interview guide included questions on perceived changes in sleep quality and experience, sleep hygiene, daily life, and overall quality of life before and after the program. The participants were also asked about their experiences with the sleep diary and their feedback on the program, including aspects they found most helpful and areas for improvement. All interviews were conducted by trained researchers, audio-recorded with the participants’ consent, and transcribed verbatim for analysis.

### Instruments

#### Sleep-related measures

##### Leeds sleep evaluation questionnaire

The Korean version of the Leeds Sleep Evaluation Questionnaire (LSEQ-K), originally developed by Parrott and Hindmarch, was adapted and validated by Kim et al.^34^^,^^35^ No significant differences were found between the original and Korean versions, with correlations confirming their equivalence.^35^

The LSEQ-K retains the original bipolar opposing statements but modifies the visual analog scale into a combined graphic and numeric scale marked at 0, 50, and 100 points. It consists of 10 items covering 4 domains: sleep initiation, sleep quality, awakening, and post-wake behavior. Higher scores indicate better perceived sleep quality. The Cronbach's alpha coefficients range from 0.78 to 0.92, confirming internal consistency and cross-cultural validity.^36^

##### Insomnia severity index

Insomnia severity was measured using the Korean version of the Insomnia Severity Index (ISI–K), translated and validated by Cho et al., in 2014 from the original scale developed by Bastien et al., in 2001.^37^^,^^38^ Briefly, the ISI is a 7-item self-report questionnaire assessing nighttime and daytime insomnia symptoms. Total scores range from 0 to 28, and higher scores indicate greater insomnia severity.^38^

##### Sleep hygiene scale

The Sleep Hygiene Scale used in this study was developed by the research team based on 11 sleep hygiene recommendations provided by the National Comprehensive Cancer Network (NCCN).^39^ The original NCCN guidelines present these items in a binary (Yes/No) format. Each item was converted into a 4-point Likert scale to quantitatively assess the participants' sleep hygiene behaviors. Higher scores indicated better sleep hygiene. The scale demonstrated acceptable internal consistency, with a Cronbach's α of 0.76 in this study.

#### Physiological measures

Cortisol and vitamin D levels were analyzed using blood samples collected by research nurses in serum separator tubes. Using a 23-gauge needle, 3–5 mL blood samples were drawn from the antecubital vein and transported to the laboratory on the day of collection. The samples were analyzed according to the manufacturer's instructions. The levels of cortisol and vitamin D were measured using the Elecsys Cortisol II assay kit (Roche Diagnostics, Meylan, France) and the JW Reagent-Vitamin D(II) on a Cobas c 702 instrument, respectively.

#### Physical measures

Forced vital capacity (FVC) and forced expiratory volume in 1 second (FEV_1_) were measured using a Pony FX spirometer (COSMED Inc., Rome, Italy). The tests were conducted three times with the participant seated, and the highest values were used for analysis.^40^

Grip strength was measured twice with the participant standing with the elbow extended. A Smedley-type dynamometer (Fabrication Enterprises Inc., Elmsford, NY, USA) was used, and the average value was used for analysis.^41^

Balance was assessed using the single-leg stance (SLS) for static balance and the Y-balance test for dynamic balance.^42^^,^^43^ Both tests were conducted twice, and the mean values were used. In the SLS assessment, the participants stood on their dominant leg with eyes open while the duration was timed.^42^ The Y-balance test measured maximum reach distances in three directions (anterior, posteromedial, and posterolateral); a composite score was calculated as the total reach divided by twice the leg length ​× ​100.^43^

Aerobic capacity was assessed using the 6-min walk test (6-MWT), recording the total distance walked along a 20-m track within 6 min.^44^

#### Psychological measures

Distress was assessed using the Distress Thermometer (DT) and Problem List, recommended tools for distress screening.^45^ Briefly, the DT measures distress over the past week on a scale of 0–10 (0 ​= ​no distress, 10 ​= ​extreme distress).^45^

Cancer-related fatigue (CRF) was measured using the Cancer Fatigue Scale (CFS), a 15-item instrument covering physical, affective, and cognitive subscales. The total scores range from 0 to 60, with higher scores indicating greater fatigue. The reliability and validity of the CFS have been demonstrated in both Japan and South Korea.^46^^,^^47^

Health-related quality of life (HRQoL) was assessed using the Korean version of the European Organization for Research and Treatment of Cancer Quality of Life Questionnaire.^48^ The 30-item instrument evaluates 5 functional dimensions, 9 symptoms, and global health status/quality of life. The scores range from 0 to 100, and higher symptom scores reflect greater symptom burden, whereas higher functional and global health scores indicate better HRQoL.

### Intervention

The forest healing program was conducted once weekly for 2 hours over 8 weeks in 2 urban forests: Incheon Grand Park and the Ansan Urban Forest. The program was collaboratively developed by a level 1-certified forest healing instructor, oncology nurse specialists, physical therapists, and the research team and incorporated evidence-based strategies to improve sleep in cancer survivors.^49^, ^50^, ^51^

Each session followed a structured format consisting of three stages: introduction, core activity, and closing. The introduction phase (“Meeting the Forest”) began with participants drinking a specially prepared herbal tea known to promote sleep, accompanied by light conversation and gentle stretching to support mental and physical transition into the forest environment.^52^

The main activities (typically 4–5 per session) varied weekly and included mindful walking, breathing and meditation exercises, nature-based games, journaling, and sensory engagement with natural materials (e.g., tree fruits, wood, and aromatic herbs). These natural materials were used to stimulate the senses and deepen the participants’ immersion in the forest environment.^53^ Sessions concluded with light stretching or group sharing to promote emotional regulation and integration.

All sessions were jointly facilitated by one level 1-certified forest healing instructor and two level 2-certified trained assistants. All the participants received consistent interventions from the same facilitators throughout the program. The program was conducted in small groups of 8–10 participants per session, and attendance was recorded at each session. Among the 29 participants, 10 participants attended all 8 sessions, 9 participants missed 1 session, 7 participants missed 2 sessions, and 3 participants missed 3 sessions. Although attendance varied, there were no dropouts. All participants completed both the pre- and post-intervention assessments and were included in the final analysis. The program structure and session-specific content are presented in Supplementary Table S1.

### Data analysis

Quantitative data were analyzed using SPSS/WIN 28.0 (version 23.0; IBM, Armonk, NY, USA). The participants’ general characteristics were summarized using frequencies, percentages, means, and standard deviations. The Shapiro–Wilk test was used to assess the normality of data distribution. Paired t-tests or Wilcoxon signed-rank tests were used to evaluate the physical and physiological changes, with significance set at *P* ​< ​0.05. Repeated measures analysis of variance with Bonferroni post hoc tests were used to analyze changes in insomnia severity, sleep hygiene, fatigue, and quality of life, with significance set at *P* ​< ​0.05. Friedman tests, followed by Wilcoxon signed-rank tests with Bonferroni correction, were used to assess distress and sleep, with significance set at *P* ​< ​0.025.

The qualitative data were analyzed using the qualitative content analysis method, guided by the frameworks proposed by Elo and Kyngäs and Lee et al.^54^^,^^55^ All semi-structured interviews were audio recorded and transcribed verbatim, and the accuracy of each transcript was verified by cross-checking it with the original audio files. The transcripts were read repeatedly to ensure familiarity with the content. Meaning units were identified, condensed, and coded and then grouped into subcategories and abstracted into overarching themes.

To ensure rigor, we applied Sandelowski's criteria:^56^ credibility, transferability, and verifiability. Credibility was strengthened through member checking with selected participants and peer debriefing among the research team. Transferability was supported by providing a detailed description of the study context, participant characteristics, and intervention process. Verifiability was maintained by keeping an audit trail of the analysis process and documenting all coding and categorization decisions.

## Results

### Results of quantitative data analysis

#### General participant characteristics

The general characteristics of the participants are presented in Table 1. All the participants were women, and the mean age was 52.1 years. Breast cancer was the most common (75.9%) type of cancer.

**Table 1:** General participant characteristics (*N* ​= ​29).

Variable	Data
Age, years	52.1 ​± ​8.7
Women	29 (100.0)
Height, cm	159.4 ​± ​5.0
Weight, kg	58.6 ​± ​13.7
BMI, kg/m^2^	23.0 ​± ​4.7
Cancer type	
Breast	22 (75.9)
Lung	2 (6.9)
Stomach	1 (3.4)
Thyroid	1 (3.4)
Colon	1 (3.4)
Cervical	2 (6.9)
Time since diagnosis, days	1106.0 ​± ​694.0
Time since treatment completion, days	590.4 ​± ​400.5

Data are presented as the median ​± ​SD or *n* (%); Percentages may not total 100% because of rounding.

BMI, body mass index; SD, standard deviation.

#### Sleep

Table 2 presents the longitudinal changes in sleep-related variables. Sleep onset time, sleep quality, behavior following wakefulness, insomnia severity, and sleep hygiene were significantly improved (*P* ​< ​0.001). Post hoc analyses confirmed significant improvements from pre-intervention to both immediately and 4 weeks post-intervention.

**Table 2:** Intervention effects on sleep (*N* ​= ​29).

Variable		T1 Mean ​± ​SD	T2 Mean ​± ​SD	T3 Mean ​± ​SD	*F/X*^*2*^	*P-value*
Sleep (LSEQ)	GTS	43.9 ​± ​19.8	70.3 ​± ​18.6	66.5 ​± ​20.6	39.11^a^	< 0.001^T1<T2,T3^^c^
	QOS	38.7 ​± ​19.8	65.1 ​± ​21.6	61.7 ​± ​24.2	20.10^a^	< 0.001^T1<T2,T3^^c^
	AFS	60.1 ​± ​28.8	68.9 ​± ​24.6	63.7 ​± ​28.0	1.45^a^	0.483
	BFW	42.4 ​± ​20.2	70.8 ​± ​21.5	64.0 ​± ​23.4	28.22^a^	< 0.001^T1<T2,T3^^c^
Insomnia (ISI)		14.8 ​± ​4.8	8.1 ​± ​4.0	9.1 ​± ​4.9	21.53^b^	< 0.001^T1>T2,T3^^d^
Sleep hygiene (sleep hygiene scale)		29.2 ​± ​4.4	32.9 ​± ​4.5	32.8 ​± ​4.7	12.89^b^	< 0.001^T1<T2,T3^^d^

T1 ​= ​Baseline (2–3 days before the first session); T2 ​= ​within 3 days post-intervention; T3 ​= ​4 weeks post-intervention.

LSEQ, Leeds Sleep Evaluation Questionnaire; GTS, getting to sleep; QOS, quality of sleep; AFS, awakening from sleep; BFW, behavior following wakefulness; SD, standard deviation; ISI, Insomnia Severity Index; SD, standard deviation.

a Friedman test.

b Repeated measures ANOVA.

c Wilcoxon signed-rank test (post hoc) with Bonferroni correction.

d Bonferroni post hoc test.

#### Psychological factors

DT scores, number of distress-related problems, CRF, and quality of life exhibited significant changes over time (*P* ​< ​0.001). Post hoc analyses revealed significant improvements from the pre-intervention period to both the immediate and 4-week post-intervention periods (Table 3).

**Table 3:** Comparison of psychological measures pre- and post-intervention (*N* ​= ​29).

Variable	T1 Mean ​± ​SD	T2 Mean ​± ​SD	T3 Mean ​± ​SD	*F/X*^*2*^	*P-value*
Distress-T	4.9 ​± ​2.1	3.5 ​± ​1.9	3.7 ​± ​2.3	15.36	< 0.001^T1>T2,T3^^a^
Distress-P	7.8 ​± ​5.3	4.7 ​± ​3.4	7.0 ​± ​5.0	21.90	< 0.001^T1,T3>T2^^a^
CRF	33.2 ​± ​9.9	23.7 ​± ​11.4	25.5 ​± ​11.4	12.40	< 0.001^T1>T2,T3^^b^
Quality of life (Korean version of the EORTC QLQ-C30)	59.1 ​± ​13.3	73.1 ​± ​14.0	70.8 ​± ​19.3	22.25	< 0.001^T1<T2,T3^^b^

T1 ​= ​Baseline (2–3 days before the first session); T2 ​= ​within 3 days post-intervention; T3 ​= ​4 weeks post-intervention.

Distress-T, Distress Temperature; Distress-P, Distress Problem List; CFS, Cancer Fatigue Scale; EORTC QLQ-C30, European Organization for Research and Treatment of Cancer Quality of Life Questionnaire 30; SD, standard deviation.

a Wilcoxon signed-rank test (post hoc) with Bonferroni correction.

b Bonferroni post hoc test.

#### Physical factors

As shown in Table 4, hand grip strength, balance ability (one-leg standing time and Y-balance test), and cardiorespiratory endurance (6-MWT) were significantly improved following forest therapy (all *P* ​< ​0.05). In contrast, no significant changes were observed in FVC and FEV_1_.

**Table 4:** Comparison of physical measures pre- and post-intervention (*N* ​= ​29).

Variable			Mean ​± ​SD	*t*(*P*) or *Z*(*P*)
**Respiratory function**	FVC	Pre	2.8 ​± ​0.5	−1.67 (0.094)^a^
		Post	2.9 ​± ​0.5	
	FEV_1_	Pre	2.3 ​± ​0.4	−1.41 (0.156)^a^
		Post	2.3 ​± ​0.4	
**Muscle strength**	Handgrip	Pre	20.5 ​± ​5.1	−3.06 (0.002)^a^
		Post	22.5 ​± ​3.9	
**Balance**	SLS	Pre	10.7 ​± ​7.7	−4.21 (< 0.001)
		Post	20.5 ​± ​16.1	
	Y-Balance	Pre	84.1 ​± ​12.8	−8.02 (< 0.001)
		Post	93.8 ​± ​10.8	
**Cardiopulmonary endurance**	6-MWT	Pre	536.6 ​± ​82.8	−2.32 (0.027)
		Post	562.2 ​± ​71.6	

FVC, forced vital capacity; FEV_1_, forced expiratory volume in 1 second; SLS, single-leg stance; 6-MWT, 6-min walk test; SD, standard deviation.

a Wilcoxon signed-rank test.

#### Physiological factors

Following the intervention, vitamin D levels were significantly decreased (*P* ​= ​0.046), whereas serum cortisol levels showed no significant changes (Table 5).

**Table 5:** Comparison of physiological measures pre- and post-intervention (*N* ​= ​29).

Variable		Mean ​± ​SD	*Z*(*P*)
Cortisol (μg/dL)	Pre	7.8 ​± ​3.6	−0.86 (0.387)
	Post	8.1 ​± ​4.5	
Vitamin D (ng/mL)	Pre	32.3 ​± ​10.1	−1.99 (0.046)
	Post	29.7 ​± ​11.2	

SD, standard deviation.

### Qualitative data analysis results

Based on the qualitative content analysis, 4 major themes and their associated subthemes were identified, as summarized in Table 6.

Theme 1 Perceived benefits of the forest.

This theme related to the participants’ experiences of the emotional and physical benefits of the forest that they newly recognized during the forest therapy program.

The participants reported discovering the therapeutic value of the forest, including the calming effects of sunlight, natural scents, and forest sounds. These sensory experiences facilitated emotional stability and physical relaxation, contributing to improved sleep quality.

“Lying on the grass under the sunlight was amazing. … I kept thinking, ‘I wish I could have more moments like this.’” (Participant 3)

“Being in nature, soaking up the sun, and feeling the calmness … it makes my body feel lighter and refreshed.” (Participant 13)

These responses reflect how direct engagement with natural elements can enhance psychological well-being and improve sleep.

Theme 2 Empathy and consolation through shared experiences.

This theme arose from the participants’ experiences of feeling a sense of kinship with the cancer survivors they met during the forest therapy program.

Engaging in group-based activities fostered strong empathy and connectivity. The participants reported finding comfort interacting with others who shared similar cancer experiences, allowing for open emotional expressions and mutual support.

“Meeting people who had gone through a similar experience allowed us to exchange emotions and talk about things I couldn’t share before.” (Participant 12)

Theme 3 Active self-management of health.

This theme depicted the behavioral changes the participants learned and adopted to take care of their own health during the forest therapy program.

The participants were motivated to manage their health proactively. They applied techniques learned during the sessions, such as breathing, stretching, and forest walking, to their daily routines and reported improvements in mood, energy levels, and sleep.

“I told myself that from now on, I need to take care of my health. I went to the mountains three or four times a week.” (Participant 2)

“I wake up in the middle of the night and practice what I learn. Doing that felt beneficial.” (Participant 13)

This illustrated a transition from passive to active engagement in personal well-being.

Theme 4 Expanded self-awareness.

This topic highlighted the participants’ awareness of their sleep habits and problems through a forest therapy program for improving sleep.

Maintaining a sleep diary encouraged the participants to reflect on their behavior and sleep patterns. This self-observation led to enhanced awareness of habits that hindered sleep and facilitated meaningful behavioral changes.

“Writing in the journal helped me track my sleeping and waking times … my quality of life improved.” (Participant 8)

“At first, I thought keeping a sleep diary was pointless. But over time, I realized patterns … Now, I want to continue keeping a sleep diary. (Participant 24)

These reflections suggested that structured self-monitoring tools, such as journaling, could foster insight and self-regulation.

Collectively, these themes suggest that the forest therapy program not only provides a natural restorative setting, but also supports psychological healing, social bonding, and self-directed behavioral change. By synthesizing the participants’ experiences into structured themes and subthemes, the analysis offers a nuanced understanding of how nature-based interventions can enhance the well-being of cancer survivors.

**Table 6:** Qualitative themes and subthemes (*N* ​= ​29).

Themes	Subthemes
**1. Perceived benefits of the forest**	1.1 Sensory engagement with nature
	1.2 Emotional and physical restoration
**2. Empathy and consolation through shared experiences**	2.1 Emotional resonance among survivors
	2.2 Nonverbal communication and mutual care
**3. Active self-management of health**	3.1 Application of learned strategies
	3.2 Motivation for lifestyle change
**4. Expanded self-awareness**	4.1 Recognition of maladaptive sleep habits
	4.2 Behavioral transformation through journaling

## Discussion

### Main findings

This study evaluated the effectiveness of an eight-session structured forest therapy program for improving multidimensional aspects of sleep, psychological well-being, and physical function among cancer survivors. The components and observed outcomes of the program were informed by and conceptually aligned with the principles of CBT-I^28^ and ERT.^29^ Both CBT-I and ERT emphasize behavioral regulation and the therapeutic effects of natural environments in supporting improvements in multidimensional aspects of sleep recovery.

Quantitative results demonstrated significant improvements in sleep-onset latency, sleep quality, and post-awakening behavior. These findings are consistent with prior studies confirming the efficacy of forest-based interventions in enhancing various aspects of sleep, including onset, quality, and post-awakening functioning, among cancer survivors.^25^^,^^54^ The integration of CBT-I elements likely contributed to these improvements, as evidenced by increased sleep hygiene scores and improved subjective sleep quality, supporting the long-term benefits of CBT-based behavioral regulation.^9^^,^^26^

The participants’ engagement in writing a sleep diary facilitated self-monitoring and provided insights into maladaptive sleep patterns, fostering behavioral change. The qualitative data further illustrated how reflective practices such as journaling and mindful forest engagement enhanced self-awareness and empowered participants to actively manage their sleep health.

The natural environment appears to support sleep recovery. Exposure to sunlight, forest aromas, and calming sounds contributes to emotional stability and physical relaxation, possibly aiding circadian rhythm regulation and melatonin secretion.^55^^,^^57^ The participants described these sensory experiences as deeply soothing, reinforcing the therapeutic value of nature-based interventions.

Both the DT scores and the number of distress-related problems improved after the intervention. Although the number of distress-related problems returned to baseline levels 4 weeks post-intervention, DT scores remained stable, suggesting an improvement in the participants’ internal coping mechanisms. This aligns with prior research on mindfulness and nature-integrated interventions that emphasize self-regulation and emotional resilience.^58^^,^^59^

CRF also decreased significantly, consistent with reports suggesting that gentle physical activity in natural settings alleviates fatigue among cancer survivors.^60^^,^^61^ The improvements in grip strength, balance, and cardiorespiratory endurance underscore the role of forest therapy as a low-intensity yet effective physical rehabilitation strategy, particularly for individuals with reduced exercise tolerance post-treatment.^60^

With respect to quality of life, both quantitative and qualitative outcomes (e.g., companionship, empathy, and shared suffering) suggest that peer bonding and emotional connectedness play key roles in enhancing psychosocial resilience and reducing isolation.^54^^,^^62^^,^^63^

The physiological markers showed mixed results. Although the changes were not significant, serum cortisol levels increased. In contrast, decreased salivary cortisol levels following forest therapy were reported in prior studies.^64^^,^^65^ Although direct comparisons are limited, these differences may reflect variations in baseline levels, measurement timing, or cortisol sensitivity to acute stress. Future research should incorporate larger sample sizes and repeated trials to examine the effects of forest therapy on serum cortisol levels.

Further, contrary to expectations based on sunlight exposure, vitamin D levels significantly decreased during the forest therapy sessions. Sun exposure increases serum vitamin D levels, and vitamin D status is associated with sleep duration.^66^, ^67^, ^68^, ^69^ Given that 75.9% of the participants in this study were breast cancer survivors, the decrease in vitamin D levels may be attributable to uncontrolled oral or injectable supplementation during the study period.^70^ Future research should monitor and control for vitamin D intake to accurately assess the independent effect of sun exposure through forest therapy on serum vitamin D levels.

### Implications for nursing practice and research

From an oncology nursing perspective, this study underscores the value of integrating nature-based and psychosocial interventions into survivorship care.^16^^,^^71^ Forest therapy offers a low-cost, non-pharmacological option that can address persistent issues such as sleep disturbances, emotional distress, and physical deconditioning. Oncology nurses are uniquely positioned to coordinate and deliver such interventions given their close contact with patients and knowledge of survivorship trajectories. These findings may provide insights for future research on the incorporation of nature-based interventions into oncology nursing education and continuing professional development, particularly within the context of integrative survivorship care.

Moreover, the structured and replicable design of this program enhances its feasibility for clinical application.^16^ Nurses can implement similar interventions in nearby urban green spaces or hospital gardens, thereby improving accessibility and continuity of care. Integrating forest therapy into existing supportive or CBT-based nursing protocols may enrich current practices in survivorship care, ultimately contributing to more holistic and patient-centered oncology nursing models.^16^^,^^72^

### Limitations

Despite these strengths, limitations must be acknowledged. The small sample size and absence of a control group reduce the generalizability of the findings. Although the intervention content remains relevant, data were collected more than 3 years ago, possibly limiting the applicability of the results to current clinical settings. The inclusion of only female participants further restricts the generalizability of the findings to a broader cancer survivor population. In addition, environmental factors, such as seasonal variation and weather conditions, may have influenced the outcomes. Future studies should recruit larger and more diverse samples and adopt controlled longitudinal designs to validate and expand upon these results.

## Conclusions

Participation in a structured forest therapy program is associated with improvements in multidimensional aspects of sleep, psychological well-being, and physical function among cancer survivors. By integrating cognitive-behavioral strategies, restorative nature exposure, and peer-based emotional support, the intervention offers a holistic approach to survivorship care. These findings highlight the potential of forest therapy as a practical, low-cost non-pharmacological nursing intervention in oncology settings. Future research should evaluate its long-term effects, scalability, and applicability in diverse populations and healthcare environments.

## CRediT authorship contribution statement

**Sang Yi Baek**: Conceptualization, Data Collection, Formal Analysis, Investigation, Writing – Original Draft; **Kwang-Hi Park**: Conceptualization, Data Collection, Supervision, Funding Acquisition; **Haneul Lee**: Conceptualization, Data Collection, Formal Analysis, Investigation, Writing – Original Draft; **Ji Hyun Sung**: Conceptualization, Data Collection, Writing – Review and Editing; **Min Kyung Song**: Conceptualization, Data Collection, Writing – Review and Editing; **Eun Young Park**: Conceptualization, Data Collection, Writing – Original Draft. All authors have read and approved the final manuscript.

## Ethics statement

The study was approved by the Institutional Review Board of Gachon University (IRB No. 1044396-202103 HR-055-03) and was conducted in accordance with the 1964 Helsinki Declaration and its later amendments or comparable ethical standards. All participants provided written informed consent.

## Data availability statement

The datasets generated and analyzed in the current study are available from the corresponding author upon reasonable request.

## Declaration of generative AI and AI-assisted technologies in the writing process

No AI tools/services were used during the preparation of this work.

## Funding

This study was supported by the R&D Program for Forest Science Technology (Grant No. 2021393A00-2123-0103), provided by the Korea Forest Service (Korea Forestry Promotion Institute). The funders had no role in considering the study design or in the collection, analysis, interpretation of data, writing of the report, or decision to submit the article for publication.

## Declaration of competing interest

The authors declare no conflict of interest.
