# Supplementary material for: Effectiveness of an urban forest healing program for improving sleep in cancer survivors
Source: Asia Pac J Oncol Nurs. 2025 Sep 29;12:100795. doi: 10.1016/j.apjon.2025.100795 (PMC12590025; doi:10.1016/j.apjon.2025.100795)
Supplement: Multimedia component 1 [file mmc1.docx]

Supplementary Table S1. Detailed Forest Healing Program for Improving Sleep in Cancer Survivors

| Session | Program | Details of activities | Time (min) |
| --- | --- | --- | --- |
| 1 | Hello? My sleep | Meet the forest (every session) | 20 |
|  |  | Make friends with the forest | 15 |
|  |  | Making natural names | 20 |
|  |  | Walking in the forest | 20 |
|  |  | Aromatherapy massage (hands) | 20 |
|  |  | Autogenic training | 15 |
|  |  | Talk about feelings (end of every session) | 10 |
| 2 | Get to know: | Map of my body | 15 |
|  | My body | Walking in the forest | 20 |
|  |  | Find a natural object that resembles me | 15 |
|  |  | Making sleeping tea | 25 |
|  |  | Autogenic training | 15 |
| 3 | Get to know: | Map of my mind | 15 |
|  | My mind | Barefoot walking in the forest | 20 |
|  |  | Aromatherapy massage (feet) | 20 |
|  |  | Sleep yoga | 15 |
|  |  | Autogenic training | 20 |
| 4 | Get to know: | Barefoot walking in the forest | 20 |
|  | You and me | Making herb smudge sticks | 20 |
|  |  | Wind bathing | 30 |
|  |  | Autogenic training | 20 |
| 5 | Worry goodbye | Slingshot shooting | 20 |
|  |  | Walking in the forest | 25 |
|  |  | Aromatherapy massage (hands) | 20 |
|  |  | Autogenic training | 25 |
| 6 | Empty mind | Stretching with wood | 15 |
|  |  | Rope play | 40 |
|  |  | Barefoot walking in the forest | 10 |
|  |  | Autogenic training | 25 |
| 7 | Refreshing body | Walking in the forest | 15 |
|  |  | Sunbathing | 15 |
|  |  | Lymphatic massage | 20 |
|  |  | Sleep yoga | 15 |
|  |  | Autogenic training | 25 |
| 8 | Welcome | Barefoot walking in the forest | 25 |
|  | good sleep | Aromatherapy massage (feet) | 20 |
|  |  | Guided imagery | 15 |
|  |  | Good sleep on my own | 30 |
